# Autoimmune Hemolytic Anemia Associated with Mature Ovarian Cystic Teratoma Containing Monoclonal Immunoglobulin G: A Case Report and Review of Literature

**DOI:** 10.1155/2024/2223281

**Published:** 2024-05-27

**Authors:** Yuma Nato, Keiki Nagaharu, Keika Itoh, Naoki Shinke, Keiko Maeyama, Akihiko Sawaki, Hiroyuki Miyashita

**Affiliations:** ^1^ Department of Hematology Yokkaichi Municipal Hospital 2-2-37 Shibata, Yokkaichi, Mie, Japan 510-8567; ^2^ Department of Hematology and Oncology Mie University Hospital 2-174 Edobashi Tsu, Mie, Japan 514-8507; ^3^ Division of Blood Transfusion Yokkaichi Municipal Hospital 2-2-37 Shibata, Yokkaichi, Mie, Japan 510-8567

## Abstract

**Background:**

Autoimmune hemolytic anemia (AIHA) associated with solid tumors such as mature cystic teratomas is rare and poorly understood. Here, we report a successfully treated case of secondary AIHA in a mature cystic teratoma containing antibodies against red blood cells. *Case description*. A 22-year-old woman was referred to our hospital with progressive anemia. Laboratory findings revealed hemolysis with a positive direct and indirect antiglobulin test. Imaging studies identified a left ovarian mass, suspected to be a mature cystic teratoma, which was later confirmed by histopathology after laparoscopic oophorocystectomy. The patient was treated with prednisolone, resulting in improved anemia. To examine the relationship between the tumor and AIHA, an indirect antiglobulin test was performed on the tumor contents. Stronger aggregations were observed at any concentration diluted by 10 times from 10 to 10,000 times of the tumor contents compared to the patient's serum. Additionally, immunofixation electrophoresis of the tumor contents revealed the presence of monoclonal immunoglobulin G-*κ*.

**Conclusion:**

The presence of monoclonal IgG-*κ* in the tumor suggests intratumoral antibody production as a possible mechanism. Further research is necessary to elucidate the pathogenic relationship between such tumors and AIHA. The report also highlights the importance of considering secondary AIHA in patients with unexplained anemia and solid tumors.

## 1. Introduction

Autoimmune hemolytic anemia (AIHA) is a condition in which the immune system produces antibodies that attack red blood cells, leading to their destruction and a reduced lifespan. AIHA stands out as one of the leading types of acquired hemolytic diseases with an incidence of approximately 1.8 per 100,000 person-years [1], and those are further subdivided into primary and secondary AIHA. Secondary AIHA frequently occurs in association with hematological cancers in 50% of cases, infections in about 33%, and collagen vascular diseases in around 17% [2]. Rare instances have also identified solid cancers, such as mature cystic teratomas, as potential triggers of secondary AIHA. Studies have indicated that secondary AIHA, when linked to mature cystic teratomas, does not typically respond well to treatments like steroids or splenectomy. However, tumor removal often leads to AIHA resolution [2]. The exact pathogenic mechanisms by which tumors induce AIHA as part of a paraneoplastic syndrome remain elusive [3]. This case report discusses the effective treatment of AIHA in a patient with a mature cystic teratoma through prednisolone administration after a laparoscopic oophorocystectomy.

## 2. Case Presentation

**Figure 1:**
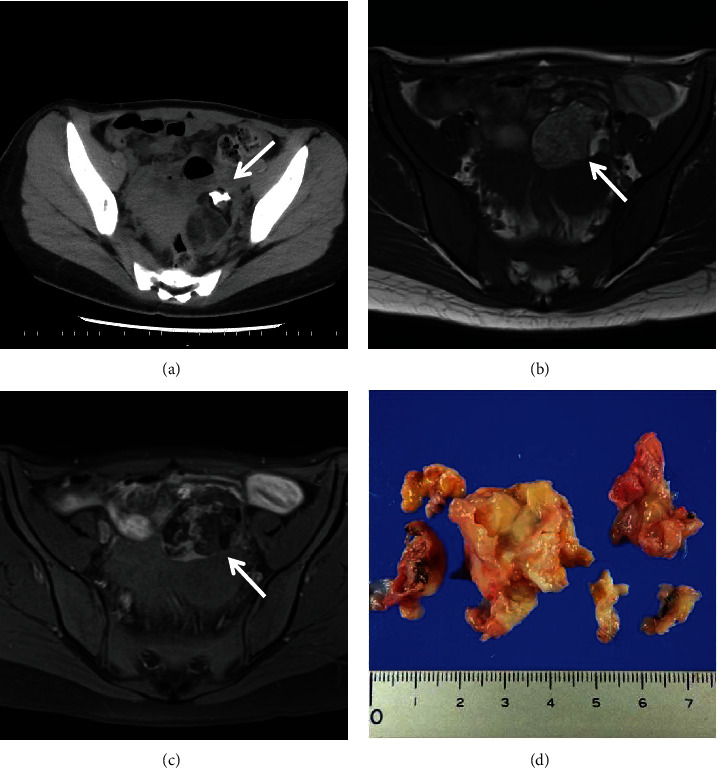
Preoperation and operation specimen. (a) Abdominal computed tomography scan on admission showed a left ovarian tumor which was 6 cm in diameter and contained high-density lesion. Magnetic resonance imaging of the abdomen at initial diagnosis: (b) the high-intensity lesion using T1-weighted images became (c) low-intensity on fat-suppression. (d)The tumor contained fluid, cartilage, skin, and skin appendage, and it was pathologically diagnosed as a mature cystic teratoma.

A 22-year-old nulliparous woman was referred to our hospital due to anemia persisting for two weeks. Laboratory tests on admission revealed a hemoglobin level of 5.5 g/dL, a circulating reticulocyte count of 280‰, a lactate dehydrogenase level of 500 IU/L (reference range, 124–224 IU/L), and an indirect bilirubin level of 1.9 mg/dL (reference range, 0.1–0.8 mg/dL). Haptoglobin was undetectable, while both direct antiglobulin test (DAT) and indirect antiglobulin test (IAT), also known as direct and indirect Coombs tests, were positive, and antinuclear antibody test was negative. Anti-e antibodies were detected upon examination of irregular antibodies. Computed tomography showed a left ovarian tumor (6 cm in diameter) without lymphadenopathy or obvious hepatosplenomegaly (Figure 1(a)). Magnetic resonance imaging revealed a suspected mature cystic teratoma (Figures 1(b) and 1(c)). In the peripheral blood sample, *α*-fetoprotein was 6 ng/mL, squamous cell carcinoma antigen was 0.5 ng/mL, carbohydrate antigen 125 was 19 U/mL, and human epididymis protein 4 was 63 pico-mol/L, respectively. These tumor markers were not elevated.

**Figure 2:**
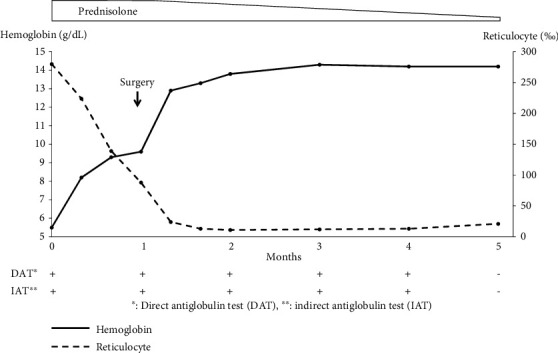
Clinical course of the disease.

The patient was diagnosed with AIHA and administered prednisolone orally at a dose of 55 mg/day, which improved their anemia. One month after initiating prednisolone therapy, laparoscopic oophorocystectomy was performed. Macroscopic examination of the tumor revealed the presence of fluid, cartilage, skin, and skin appendages. The tumor was pathologically diagnosed as a mature cystic teratoma (Figure 1(d)), and microscopic examination did not reveal any cells indicative of hematological malignancies within the tumor. The patient's anemia was normalized postoperatively without the need for blood transfusion. Four months after surgery, both the DAT and IAT results were negative (Figure 2).

We hypothesized that antibodies would be present in the fluid layer and, therefore, examined it. Additional studies were performed to clarify the relationship between tumors and AIHA.

### 2.1. Study Samples

After obtaining informed consent, laparoscopic oophorocystectomy was performed, and the mature cystic teratoma was resected. During the surgery, intratumor contents were extracted under sterile conditions. The tumor liquid contents were then centrifuged at 3000 g for 5 min at room temperature twice to remove impurities. The sample supernatant was used for further analysis.

### 2.2. IAT of the Mature Cystic Teratoma Contents

To perform the IAT on the tumor contents, the serous contents of mature cystic teratoma and the patient's serum were diluted with normal saline as 1 : 10, 1 : 100, 1 : 1000, and 1 : 10000. Ten percent of group O RBCs were incubated with the tumor fluid or patient's serum at 37.0°C for 30 minutes. After incubation, the treated RBCs were washed three times with normal saline. The IAT was performed using a polyclonal anti-immunoglobulin G (IgG) antibody, and any irregular antibodies were examined using a Data-Cyte Plus kit (Grifols, Barcelona, Spain).

### 2.3. Results

**Figure 3:**
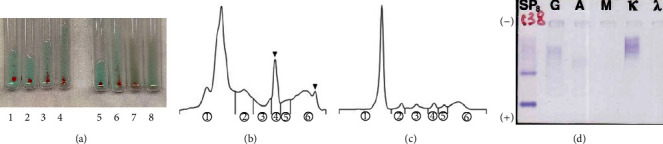
Additional laboratory investigation. (a) The lanes indicate albumin, *α*1 globulin, *α*2 globulin, *β*1 globulin, *β*2 globulin, and *γ* globulin from left. IAT of the mature cystic teratoma contents revealed stronger aggregation observed at all concentrations of the tumor contents (ranging from 10 to 10,000) compared with the patient's serum. Lanes 1 to 4 show the IAT of the mature cystic teratoma contents, and lanes 5 to 8 present that of the patient's serum. Lanes 1 to 4, or 5 to 8 indicate the dilution with normal saline as 1 : 10, 1 : 100, 1 : 1000, and 1 : 10000 from left, respectively. (b) The protein fraction of the tumor contents showed an increase in the *γ*-globulin and *β*1-globulin fraction; (c) no increase was observed in the *γ*-globulin and *β*1-globulin fraction of the patient's serum harvested before. (d) Immunofixation electrophoresis of the tumor contents showed IgG-*κ* M protein.

The IAT performed on the contents of the mature cystic teratoma showed agglutination at all tested dilutions of the tumor extract (ranging from 1 : 10 to 1 : 10,000), a reaction more pronounced than that observed with the patient's own serum (as depicted in Figure 3(a)). When screening for irregular antibodies within the tumor, there was no reactivity to anti-e antibodies.

Capillary electrophoresis was employed on both the tumor extract and the patient's serum collected before any treatment. The protein fraction of the tumor contents showed an increase in the *β*1-globulin fraction and a slight increase in the *γ*-globulin fraction (Figure 3(b)), which was not observed in the patient's serum harvested before treatment (Figure 3(c)). Furthermore, immunofixation electrophoresis of the tumor contents revealed monoclonal antibodies of IgG-*κ* type (Figure 3(d)).

## 3. Discussion

In this report, we described a case of secondary AIHA effectively managed in a patient with mature cystic teratomas.

Although there have been case reports of AIHA associated with mature cystic teratoma in recent years [4–6], the exact mechanism between mature cystic teratoma and AIHA remains elusive. However, several theories have been proposed regarding the etiology of secondary AIHA associated with mature cystic teratomas, including (1) immune responses against abnormal RBCs; (2) changes in RBC antigenicity caused by tumor-derived substances, like haptens; and (3) in situ antibody production within the tumor tissue, possibly by lymphoid cells [7].

Our literature review uncovered 12 previous cases of secondary AIHA linked to mature cystic teratomas where the IAT on intratumor contents (tumoral-IAT) was conducted (Table 1) [7–17]. Previous cases of secondary AIHA associated with mature cystic teratomas containing lymphocytes or plasma cells within the tumor cyst support the third theory [7, 11]. Besides, it was noted that patients with positive tumoral-IAT results demonstrated an 83% response to steroid treatment, which was notably higher than the 40% response rate in patients with negative tumoral-IAT. Also, the effectiveness of surgical removal of the tumor implies that the tumor tissue itself possesses the causative key for AIHA occurrence. Thus, in the case of positive tumoral-IAT, the pathophysiology of AIHA associated with mature cystic teratoma might be explained by in situ antibody production within the tumor tissue. There is a possibility that steroid therapy followed by tumor resection may be effective.

Furthermore, our case is notable due to the comparatively higher titer of tumoral-IAT than serum-IAT and the detection of monoclonal IgG within the tumor fluid. Although our examination did not reveal any hematological malignancies, the heightened agglutination in the tumoral-IAT and the presence of monoclonal IgG within the tumor strengthen the theory of intratumoral lymphocytes' role in anti-RBC antibody production. Further investigative work is needed to clarify the precise mechanisms at play in secondary AIHA.

## Figures and Tables

**Table 1:** Reported cases of AIHA secondary to mature cystic teratoma wherein IAT of the tumor content was performed.

	Age (years)	IAT of the tumor contents (type)	Efficacy of steroids	Efficacy of splenectomy	Efficacy of cystectomy	Period until serum IAT becomes negative after surgery (month(s))
Allibone and Collins (1951) [8]	4	—	not done	Not done	+	Unknown
Sandøe (1953) [16]	30	+(unknown)	+	Not done	+	2
Andre and Dreyfus (1955) [9]	54	—	—	Not done	+	Unknown
Muller and Schubothe (1958) [14]	44	—	—	—	+	4
De Bruyère et al. (1971) [7]	32	+(unknown)	—	±	+	Unknown
Buñuel et al. (1976) [10]	47	+(unknown)	±	Not done	+	3
Payne et al. (1981) [15]	34	+(IgG)	±	Not done	+	1
Buonanno et al. (1984) [11]	13	+(IgG)	+	Not done	+	3
Ikeda et al. (1985) [13]	40	—	±	Not done	+	3
Suzuki et al. (1993) [17]	20	—	—	Not done	+	3 to 4
Cobo et al. (1996) [121]	37	—	+	Not done	+	3
Present case (2022)	22	+(unknown)	+	Not done	+	4

## Data Availability

For original data, please contact keiki-nagaharu@med.mie-u.ac.jp.
